# Impact of Graphene Derivatives as Artificial Extracellular Matrices on Mesenchymal Stem Cells

**DOI:** 10.3390/molecules27020379

**Published:** 2022-01-07

**Authors:** Rabia Ikram, Shamsul Azlin Ahmad Shamsuddin, Badrul Mohamed Jan, Muhammad Abdul Qadir, George Kenanakis, Minas M. Stylianakis, Spiros H. Anastasiadis

**Affiliations:** 1 Department of Chemical Engineering, University of Malaya, Kuala Lumpur 50603, Malaysia; 2 Institute of Biological Sciences, Faculty of Science, University of Malaya, Kuala Lumpur 50603, Malaysia; shamsulshamsuddin@um.edu.my; 3 Institute of Chemistry, University of the Punjab, Lahore 54590, Pakistan; mabdulqadir@gmail.com; 4 Institute of Electronic Structure and Laser, Foundation for Research and Technology-Hellas, GR-70013 Heraklion, Greece; gkenanak@iesl.forth.gr (G.K.); spiros@iesl.forth.gr (S.H.A.); 5 Department of Nursing, Faculty of Health Sciences, Hellenic Mediterranean University, GR-71410 Heraklion, Greece

**Keywords:** nanotechnology, graphene oxide, mesenchymal stem cells, tissue engineering

## Abstract

Thanks to stem cells’ capability to differentiate into multiple cell types, damaged human tissues and organs can be rapidly well-repaired. Therefore, their applicability in the emerging field of regenerative medicine can be further expanded, serving as a promising multifunctional tool for tissue engineering, treatments for various diseases, and other biomedical applications as well. However, the differentiation and survival of the stem cells into specific lineages is crucial to be exclusively controlled. In this frame, growth factors and chemical agents are utilized to stimulate and adjust proliferation and differentiation of the stem cells, although challenges related with degradation, side effects, and high cost should be overcome. Owing to their unique physicochemical and biological properties, graphene-based nanomaterials have been widely used as scaffolds to manipulate stem cell growth and differentiation potential. Herein, we provide the most recent research progress in mesenchymal stem cells (MSCs) growth, differentiation and function utilizing graphene derivatives as extracellular scaffolds. The interaction of graphene derivatives in human and rat MSCs has been also evaluated. Graphene-based nanomaterials are biocompatible, exhibiting a great potential applicability in stem-cell-mediated regenerative medicine as they may promote the behaviour control of the stem cells. Finally, the challenges, prospects and future trends in the field are discussed.

## 1. Introduction

Over the years, stem cells have evidenced great potential for tissue regeneration and repair due to their capability to differentiate into specialised adult cell types, as well as to be indefinitely self-renewed [1]. Unfortunately, the option for sustainable renaissance also occurs for malignant growth cells, which are divided under a disruptive manner, contrary to the highly controlled stem cells proliferation [2]. Thus, the regulation of the stem cell’s fate from becoming cancerous and dysfunctional during regeneration, through tissue engineering and other biomedical approaches, remains a challenge [3]. In addition, the behaviour of a stem cell in terms of normal cellular rejuvenation and other tissue functions is strongly influenced by several factors, such as substrate topography (ST), extracellular matrix (ECM), and stem cell-substrate interactions [4]. Essentially, the integrated structure of stem cells is comprised of an ECM with built-in niche cells. The ECM is a multifunctional network consisting of a fibrous, gel-like material that surrounds the stem cell, involved in the mediation of its fate [5]. Therefore, understanding the interactions between a stem cell and its ECM is still an ongoing challenge.

ECM signalling in a niche stem cell has been recently found to be very critical since a regular stem cell fate is provided, while endogenous stem cell repair and development of synthetic ECM scaffolds for therapeutic targets have been encouraged [6]. Furthermore, various approaches for tissue regeneration, such as gene and cell therapy, and cytokine or growth factors therapy, necessitate the use of a scaffold in order to properly retain cells or cytokines and to generate an adequate number of new tissues [7]. In this frame, many studies demonstrated the role of collagen as an ECM for tissue regeneration, although it is associated with a xenogeneic immune rejection. Therefore, novel human-safe synthetic biocompatible polymers to serve as scaffolds in tissue regeneration should be developed [8].

Nanotechnology is an emerging and very promising field of science which explores several biological and techno-industrial systems. The exploitation of graphene-based nanomaterials has motivated a rapid development with respect to the reinforcement of the current technology of structured bio-nanomaterials in tissue engineering [9,10]. Graphene as a single-atom thick sheet of sp^2^ hybridized carbon atoms is well-known for its remarkable properties, such as excellent mechanical strength, thermal and electrical conductivity, and high surface area [11,12]. Moreover, graphene oxide (GO) is obtained upon the oxidation of graphite through well-known oxidation processes, resulting in the attachment of oxygen functional groups onto the basal plane and the edges of the flakes [13]. In 1859, Benjamin de Brodie was the first to synthesize GO by oxidising and exfoliating natural crystalline graphite [14]. It should be noted that nowadays, when “The Rise of Graphene epoque” is running, the “old-fashioned” de Brodie’s study came again to the fore, after almost one and a half centuries. In this frame, it is recommended as one of the most efficient and low-cost methods to prepare GO; a highly intriguing and promising material [15].

Although the structure of GO is very similar to graphene, its properties significantly differ, including lack of visible light absorption, low electrical conductivity, and much higher chemical activity than graphene, as well [16]. Due to these unique characteristics, the exploitation of graphene and GO in various applications, including the biomedical ones, has been intensively explored [17]. Moreover, GO has displayed great potential to bind growth factors in stem cell differentiation, since it may serve as a carrier as in the case of mouse embryonic stem cells (ESCs) differentiation to dopamine neurons in haematopoietic lineages [18].

In addition, GO nanoparticles (NPs) find it much easier to penetrate the cell membrane and thus they are considered as ideal biocompatible and mechanically stable components to support the growth and differentiation of the stem cells [19]. Kang et al. investigated the effects of various carbon allotropes, such as GO, carbon nanotubes (CNTs) and graphene, on dopamine neural differentiation of mouse ESCs [20]. It was confirmed that only GO could effectively promote the differentiation of dopamine neurons following a typical stromal cell-derived inducing activity (SDIA), while the dopamine neuron-related gene expression was significantly increased. Therefore, they concluded and proposed that the potential use of GO as a nanoplatform could enable the differentiation of dopamine neural ESCs, thus exhibiting a great potential applicability in cell transplantation therapy [21].

In a similar frame, Halim et al. investigated the role of GO in fostering embryonic stem cells differentiation in the haematopoietic lineage [22]. They discovered that GO-coated substrates significantly contributed to the differentiation degree enhancement of mouse ESCs in both primitive and definitive haematopoietic cells used in this study. On the one hand, GO promoted the transition of haemangioblasts to haemogenic endothelial cells, a critical step in haematopoietic specification [23]. On the other hand, GO presented an improved human ESCs differentiation to blood cells compared to the case of testing murine ones. To summarize, the study demonstrated the beneficial role of GO towards haematopoietic differentiation, while as a viable backup plan, a large number of functional blood cells could be generated, upon a specified functionalization of GO [24].

More recently, the incorporation of GO in stem cells significantly facilitates binding, proliferation, and differentiation in osteogenic and myoblast cells, as seen in the case of MSCs [25]. Due to its honeycomb structure, it could serve as an outstanding artificial extracellular matrix [26]. In this context, GO-doped poly(lactic-co-glycolic acid) (PLGA) nanofibers prepared via electrospinning were exploited to fabricate novel, highly biocompatible scaffolds [27]. The potential applicability of GO was explored and compared with one of the other nanomaterials, such as CNTs, to develop platforms ideal for cell culture. It was proved that among the most conventional nanomaterials, GO is the most promising one to promote the growth and differentiation of the stem cells [28].

Even though graphene is considered as a potential material for biomedical applications, there are only a few studies in the literature where its role and impact on the human body have been exclusively interpreted [29]. Compared to graphene, GO exhibits moderate toxicity, while it may cause a partial cell growth inhibition. However, at high dosages (~50 mg/L) it may slightly delay the growth of some cell types, as in the case of zebrafish embryos [30]. In contrast, at a relatively low concentration (25 mg/L), multiwalled carbon nanotubes (MWCNTs) exhibit acute toxicity, thus inhibiting cell proliferation and causing serious morphological flaws in the embryonic hatching [30]. However, previous experiments revealed no cytotoxicity during the in vitro experiments of HCT-16 cells when conducted with PEG functionalized graphene [31]. Although recent studies elaborated the influence of GO as a promising regenerative material for skin, tissues, bones and nerves, its impact has been scarcely analysed, particularly on the MSCs [32]. In this review article, we highlight the role and impact of GO as an artificial ECM on MSCs mediated treatments, summarizing the most recent studies on the topic. We also discuss and compare its biocompatibility on human and rat MSCs, while the challenges and potential applicability of graphene derivatives in the field of regenerative medicine are considered.

## 2. Stem Cells and Potential Differentiation of Growth Factors

Owing to the multifunctionality and different properties of the stem cells, their description remains complicated. Focusing on the differentiation of a single cell, stem cells can be better defined by taking into account their common properties and characteristics; (a) they do not fully differentiate and (b) they are capable of discrimination into multiple mature cell types while their characteristics are retained [33]. Therefore, stem cell potency is highly important. For instance, pluripotent cells can differentiate into ectoderm, mesoderm, and endoderm, being transformed into any cell type of the body, contrary to multipotent cells, which only segregate into closely related cell types [30,34].

Depending on their potency, stem cells can replace specialised cells which have been damaged, lost, or died. They can be either indefinitely divided to generate new cells or be transformed into other cell types depending on the body needs [35].

Stem cells can be met in various types, including: (1) ESCs (pluripotent)—they may differentiate into three germ layers without losing their pluripotency; (2) induced pluripotent stem cells (IPSCs) (pluripotent)–pluripotency-related genes are introduced into the genome of matured, fully differentiated fibroblasts in the lab and (3) multipotent MSCs-adult stem cells that keep on regenerating and differentiating into specialised cells [36,37]. Stem cells have been used in research in order to better understand the cell basis before, during, and after the treatment of a disease. Furthermore, stem cells enable the replacement of lost or damaged cells in the body which are impossible to be regenerated in a natural manner [38]. A deeper understanding of the ESCs growth is essential to fully comprehend the stem cell’s nature, in order for the type of differentiation to be regulated. On the other hand, pluripotent stem cells are difficult to grow in the lab and more scarcely in body parts, despite their greater option and capability to resolve DNA related issues [39].

**Figure 1:**
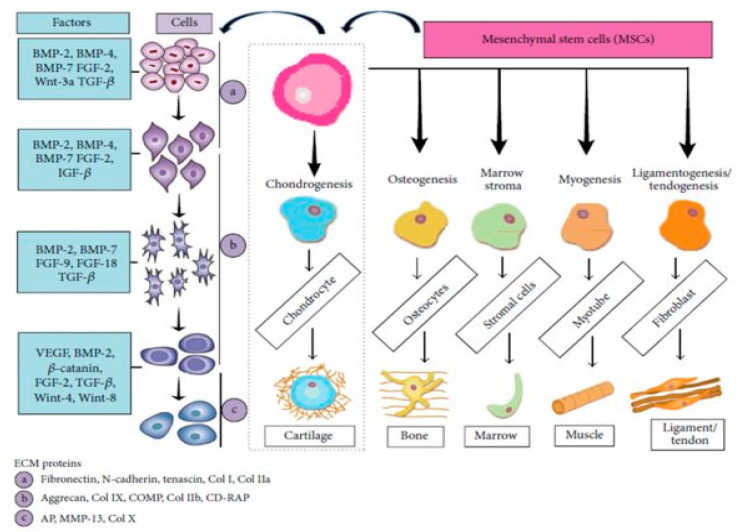
MSCs differentiation types and growth factors of various cell types. Illustration of ECM proteins at various stages [41].

Growth factor is a protein/peptide produced by various cell types which regulates the growth of target tissues and cellular potential proliferation, and/or differentiation, as well. Each growth factor has a unique cell-surface receptor which transfers growth signals to other intracellular components, resulting in the initiation or inhibition of the cell division and gene expression, respectively, as presented in Figure 1 [40,41].

Growth factors and chemical agents have been frequently used to promote stem cell proliferation and differentiation. In this frame, researchers working on stem cell biology previously utilized appropriate growth factors to stimulate proliferation, differentiation and/or migration of the stem cells [42]. Despite the fact that embryonic pluripotent stem cells have an infinite capacity for self-renewal and differentiation into three germ layers, ethical concerns on their use favoured the development of IPSCs [43]. Thus, due to the impact of growth factors on IPSCs variation, the possibility to establish an infinite supply of embryonic-like stem cells enhances. Unfortunately, this approach presents several drawbacks, including adverse side effects, degradation, denaturation, and high cost [44]. To overcome these obstacles, several graphene-based nanomaterials have been recently investigated and incorporated into various biomedical applications to provide a better initiative, in order to better control the behaviour of the stem cells [45].

## 3. Graphene and Graphene Oxide

Graphene has an overabundance of potential applications in various fields of interest, including optoelectronics, photonics, medicine, and so on [46,47]. For instance, nonlinear optical materials have been widely exploited towards the protection of sensitive instruments from laser-induced damage [48], to increase the performance in water treatment technologies [49], to form functional coatings (e.g., graphene coated plastic materials which are used to improve shelf life in medical firms and to increase resistance against corrosive acids) [50], to improve conductivity in rechargeable batteries [51], to prepare lenses with improved microscopic refractive indices by reducing the thickness of the lens [52], and to store hydrogen for medical purposes [53].

Graphene is one of the most promising materials in nanotechnology due to its exceptional electronic, thermal, mechanical, and optical properties [54]. For the first time, graphene was isolated through the mechanical exfoliation of graphite in 2004 [55]. As previously mentioned, graphene can be synthesised according to a wide range of top-down and bottom-up techniques [56]. Despite its thinness and light weight, it exhibits an incredible strength (it is considered as the strongest material), flexibility and stretchability [57]. It should be also noted that graphene and its derivatives exhibit great biological properties and thus they have been extensively utilized in various biomedical applications [58]. In addition, graphene derivatives can interact with various biological molecules, such as proteins and nucleic acids, thus they may influence toxicity. This interaction primarily affects the physical properties of the molecule and may result in human body impairment, including the damage of cells, tissues, and even organs [59]. Furthermore, graphene exhibits excellent antibacterial activity due to its physiochemical properties, and therefore recent comprehensive studies have reported on its incorporation in biomedical and human health related applications [60,61,62].

Except from graphene, many of its derivatives have been widely used in such applications since they display similar or complementary properties and characteristics [44,60]. Thus, selective modification to develop graphene-based nanomaterials with desirable and appropriate properties is required in order to completely control their operational role and compatibility depending on the application of interest. Among the numerous graphene derivatives, GO and its reduced form (reduced graphene oxide-rGO) are the most common, mainly due to the presence of oxygen functional groups, which can serve as possible active sites to enable further functionalization [63].

**Figure 2:**
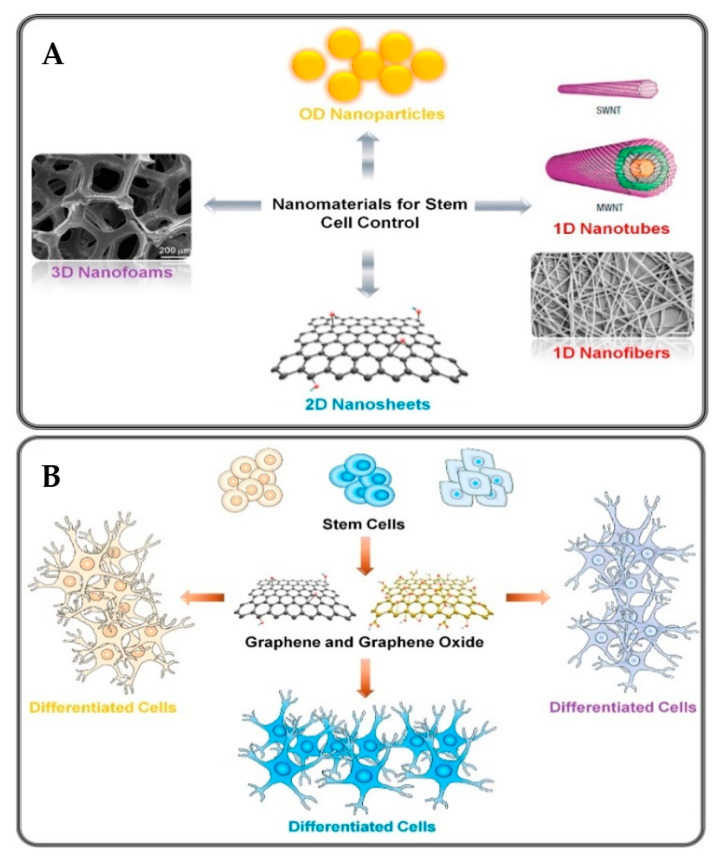
Selective nanomaterials and stem cell growth. (**A**) Potential carbon allotropes for stem cell differentiation and growth, (**B**) the effect of graphene and GO on stem cell growth, proliferation, and tissue lineages [10].

The lack of cell–cell interactions in conventional stem cell differentiation has led in the utilization of GO to provide cell-adhesion for the culture of stem cells in regenerative medicine [64]. Since GO is fluorescent, it is particularly appropriate for various biomedical applications, such as biosensing and the detection of other diseases [65]. These graphene-based biomaterials have been utilized as cell-adhesion substrates, growth factors and differentiation protein-delivery carriers to assist the differentiation of adult chondrogenic stem cells, as depicted in Figure 2 [66].

MSCs originating in the bone marrow, cord blood, peripheral blood, foetal liver, and lung, are characteristic examples of multipotent stem cells [67]. They are considered as multipotent due to their ability to differentiate into a number of cell types, such as adipocytes, osteoblasts, chondrocytes, and myocytes [68]. Owing to the potential and ease of growth in cell culture, these adult stem cells have been widely investigated [69]. MSCs play a key role in tissue regeneration thanks to their capability of migration to the sites of injury in order to replace the dysfunctional cells. They direct a variability of chemokines and cytokines which boost healing of degraded tissues and restoration of metabolism and inflammation. Usually, the emission of therapeutic factors enhances upon permission by inflammatory signals or apoptosis, which are encouraged by the host immune system [70].

**Figure 3:**
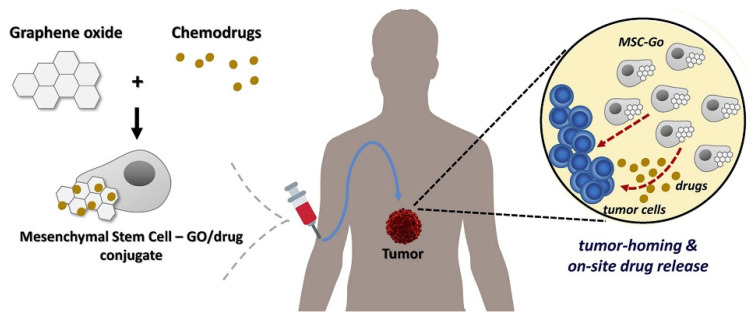
Schematic representation of GO as a competent drug nanocarrier on MSCs for cancer therapy [74].

**Figure 4:**
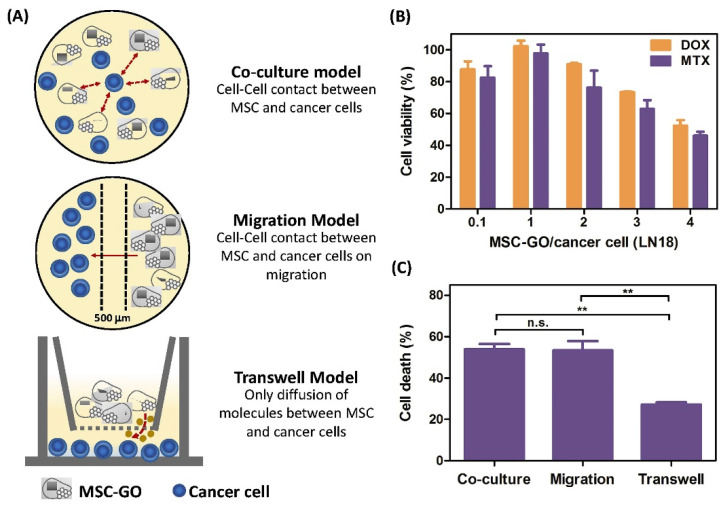
The effects of GO based drug delivery systems on MSCs: (**A**) The function of three different toxicity models, (**B**) the assessment of cancer cell (LN18) viability upon the loading of two different drugs in various amounts, (**C**) cancer cell (LN18) toxicity evaluation of the three models presented in (**A**) (where n = 3; significance: ** *p* < 0.01; n.s.: not significant; determined by One-way ANOVA) [74].

MSCs have shown great effectiveness on many cell-mediated therapies. During treatment, the cells can be originated from allogeneic (from a different person) or autologous (from the same person) sources [71]. The potential of tissue replacement is directly related to the number of risks and obstacles that must be overcome to develop novel therapeutic strategies in the case of cell-mediated treatments [72]. In this regard, an effective cancer therapy was introduced, based on the loading of GO on MSCs, which served as excellent nanocarriers (Figure 3 and Figure 4) [73].

The demand for further improvement in stem cell adhesion, growth and differentiation is an important aspect that has emerged the use of GO in the field [75]. MSCs are capable of self-renewal and differentiation into various types of cells, originated by the bone marrow, adipose tissue, dermis, and other tissues. Therefore, they could serve as excellent platforms towards the advance of the biomedical field for regenerative medicine, tissue protection and immunomodulation related applications [76]. Since GO presents many extraordinary properties (i.e., strength, conductivity, transparency, etc.), it is considered as an ideal biocompatible model to explore the interactions which take place in the MSCs [77]. In this regard, GO has been widely utilized as a scaffold for stem cell growth and proliferation in urothelial surgeries, cardiac surgeries, as well as a carrier in drug delivery systems [78]. Due to the growing interest for stem cells incorporating GO-based nanomaterials, many studies have been conducted to comprehend and analyse the toxicity, biodegradability, and biocompatibility of such complex systems [79,80].

In addition, the use of GO as a scaffold has greatly enhanced the interactions between cells and their surroundings [81]. In this frame, the interactions between the stem cells and GO based scaffolds have been momentously studied, and therefore advanced knowledge on the behaviour of stem cells has been acquired. Undoubtedly, the culture and differentiation of the stem cells are strongly dependent on the structure, size, and properties of GO [61,62,82,83].

Huynh et al. investigated the performance of a novel polymer-coated GO based drug carrier [84]. Due to the advantageous dispersibility of GO in aqueous media compared to graphene, it is more applicable for biological purposes [85]. In general, 2D and 3D structured graphene derivatives with appropriate biocompatibility, morphology, versatile chemical states, high physicochemical stability, suitable flexibility, and in vivo degradation capability, have presented great potential applicability towards the acceleration, adjustment, and control of the stem cell differentiation into specific lineages [86]. On top of that, graphene-based nanomaterials have played an important multi-role in the research of stem cells, serving either as growth substrates or tissue scaffolds, as well as intra- and/or extracellular matrices [87].

GO can be effectively used as tissue engineering scaffold owing to its extraordinary mechanical properties and the capability to customise various functionalities on flat surfaces. Therefore, it may highly encourage the differentiation of human MSCs into the osteogenic lineage [88]. In addition, GO serves as protective coating for implants in bone tissue engineering, being an ideal scaffold for in vivo bone tissue regeneration [89]. In a similar trend, the cardiac differentiation of human ESCs on graphene substrates can be also improved, mainly due to the roughness of the graphene-based coating.

Overall, human MSCs cultured on graphene- and GO-coated surfaces exhibited an accelerated cell adhesion, proliferation and differentiation compared to the ones cultured on polydimethylsiloxane (PDMS), polyethylene terephthalate (PET), glass, silicon, or silicon dioxide substrates [90,91]. It is apparent that GO enables the pluripotency of IPSCs, which spontaneously differentiate into embryonic bodies to distinguish into various cell types. Hence, GO may surprisingly provide a simple, low-cost, and reproducible method for preserving the pluripotency of IPSCs for stem cell therapy and tissue engineering applications [92].

In another case study, the differentiation of neural stem cells (NSCs) was significantly improved onto graphene-based substrates compared to the reference glass slides [93]. It was confirmed that the stem cells cultured on graphene-based coatings could differentiate into more neurons and fewer glia cells, while the adhesion was much better than in the case of glass slides [94]. Moreover, the utilization of biocompatible ginseng-graphene showed great potential in the differentiation of human NSCs into neural cells, as demonstrated in another study by Akhavan et al. [95].

**Figure 5:**
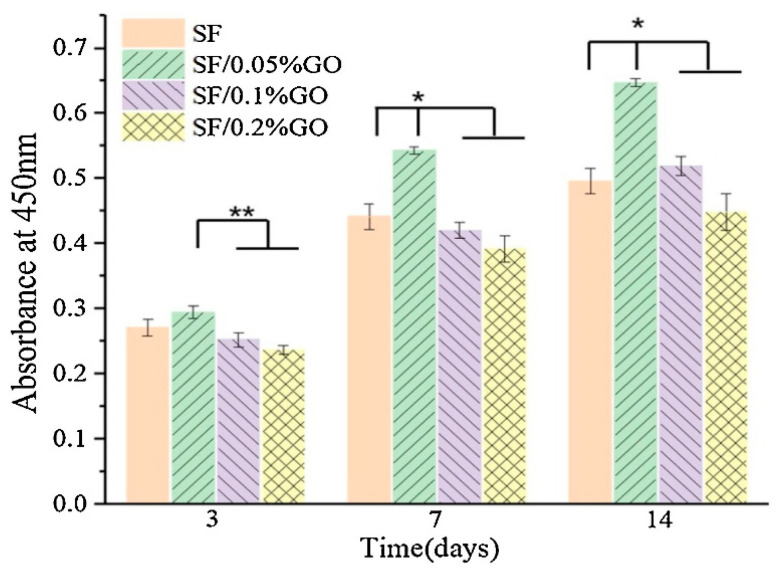
MSCs proliferation using SF/GO composites in different ratios, i.e., SF/0.05% GO, SF/0.1 %GO, and SF/0.2% GO. Proliferation rate was indirectly estimated by the absorbance as a function of time (** *p* < 0.05, * *p* < 0.01) [98].

In order to deliver cells or growth factors to an injured site, a stem cell-based therapy in regenerative medicine and tissue engineering frequently requires the presence of a scaffold. Due to non-toxic and effective cell proliferation, GO and silk fibroin were used as promising biomaterials to perform a CCK-8 test and examine the cell viability and proliferation, as shown in Figure 5 [96]. Each group displayed a growing trend during the first 14 days of the culture tests. More specifically, the samples did not present any significant difference until the 3rd day, while after the 7th and 14th day, a high proliferation rate was detected. It should be noted that the maximum absorbance at 450 nm corresponded to the composite SF/0.05%GO [97,98].

## 4. Cytotoxic Activity of Graphene-Based Nanomaterials

To date, the toxicity of graphene derivatives has been considered after extensive in vivo and in vitro studies, confirming that it highly depends on their structure, morphology, and properties, which can be adjusted through functionalization [99]. Researchers have spotted an opposite effect on viscosity (and fibre diameter) with GO-grafted PEG. The filler functionalization increases fibre diameter, dispersion, and improved interface area. Further, covalent modification with polyethylene glycol (PEGylation) can reduce cytotoxicity, and hence improve the biocompatibility and stability [100]. To evaluate the toxicity of these nanomaterials, mammalian cells have been most commonly used in research centres and labs proving that graphene derivatives such as GO and rGO are particularly cytotoxic and genotoxic to cells [101].

### 4.1. Graphene Family Combined with Human Mesenchymal Stem Cells

As mentioned, the usage of graphene-based materials to serve as scaffolds have provided greater therapeutic benefits among the MSCs [102]. The scaffold consists of a porous network where cells can be attached in order to receive nutrients. It has been found that graphene derivatives may serve as promising dual role biocompatible scaffolds since they may: (a) boost differentiation of human MSCs into bone cells and (b) inhibit proliferation [100,103].

Some scaffolds are designed to encourage cell growth in a cultured environment and to host MSCs displaying a significant improvement in cell proliferation, collagen deposition, and new bone formation [104]. In the case of tissue growth, the scaffold must be highly porous with a large surface area in order to allow the transfer of nutrients to cells [105]. In contrast, a scaffold-free cell sheet is critical for stem cell-mediated tissue regeneration. The use of biomaterials-based scaffolds in conjunction with living stem cells for tissue regeneration is a leading tissue engineering approach [106]. Likewise, the MSCs are suitable for musculoskeletal tissue regeneration due to the capability of differentiation into specific tissues, such as bone, muscle, and cartilage. Hence, the regeneration efficiency can be enhanced by successfully directing the fate of the MSCs via factors and inductors [107].

**Figure 6:**
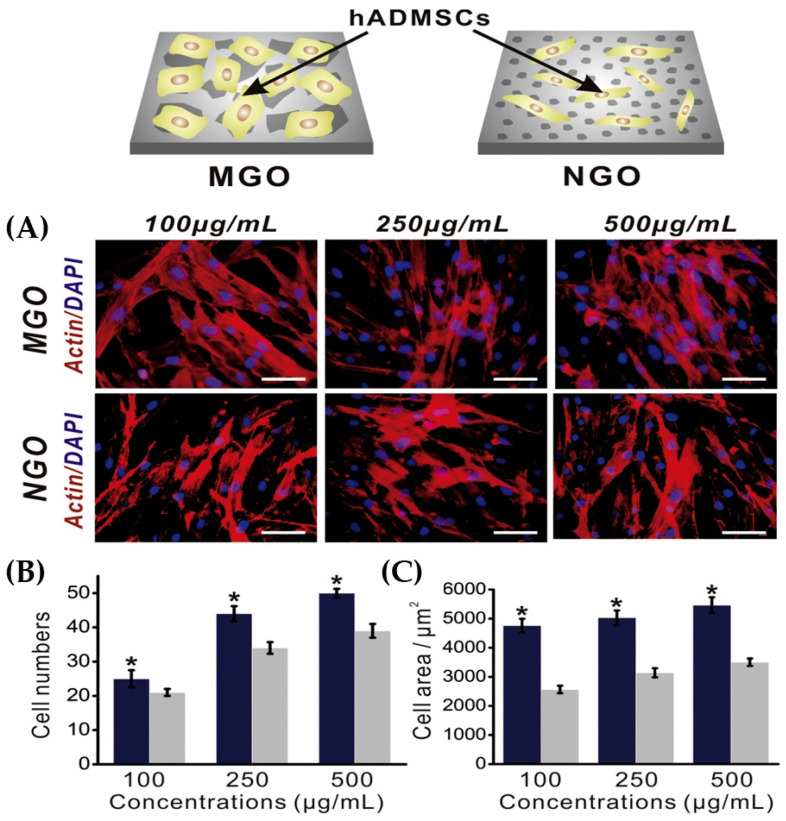
Size effects on human adipose-derived MSCs using micro and nano-sized GO substrates presenting enhanced proliferation rates of 100, 250 and 500 μg/mL (**A**) 2 days of proliferation, (**B**) cell population and (**C**) proliferated cell areas after 2 days of proliferation (* *p* < 0.05, n = 3; navy: MGO, gray: NGO) [111].

According to the literature, low doses of graphene-based materials are safe and nontoxic, as they can promote cell division [108]. Although their physiochemical properties undoubtedly influence toxicity in biological systems, there is still high demand for an integrated evaluation of the toxicity of such materials. It should be noted that the presence of any contaminant/by-product originated by the usage of graphene-based materials could also affect toxicity [109]. Numerous manufacturing methods can be applied to prepare graphene derivatives with well-defined size, shape, and surface morphology [110]. Due to these morphological characteristics, the impact of nano and micro-GO materials (NGO/MGO) on human adipose-derived MSCs have been evaluated, confirming that toxicity was strongly affected, as displayed in Figure 6 [111,112].

Table 1 summarizes the toxicity evaluation notes upon exposure of different cell types to various graphene-based materials as listed in Table 1.

Since GO endows special properties when combined with metal oxide NPs, it is an additional reason that has gained an increasing attention in the last years. Overall, the presence of graphene-based nanostructures can protect the stem cells against apoptosis, thus extending their lifetime [118], while their combination with MSCs has resulted in very low cytotoxicity [119]. In a recent study, different strategies towards the improvement and acceleration of data mining to analyse the cytotoxic potential of graphene and its physiochemical properties has been attempted. For example, machine learning has been employed to study the cell model using experimental parameters which induce cytotoxicity [120]. Furthermore, an increasing number of research groups devoted their attention to develop alternative MSCs mediated therapies. In this framework, many articles have reported on the combination of various approaches to resolve any resulted synergistic effect on cytotoxicity which remains a major clinical problem in tissue engineering [115,119,121].

**Figure 7:**
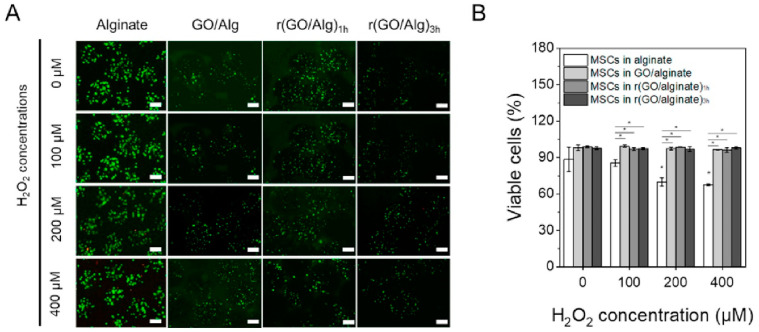
Human MSCs interactions with numerous types of microgels, i.e., Alginate, GO/Alg, rGO/Alg (1 h) and rGO/Alg (3 h). (**A**) Screening of stains at various H_2_O_2_ concentrations, (**B**) the microgels’ percentage content incubated in MCSc using different H_2_O_2_ concentrations, where existed a remarkable transformation (* *p* < 0.05) [124].

Due to the presence of various oxygen functional groups, GO is highly hydrophilic and dispersible in water, while it can be easily functionalized or decorated with various (bio)molecules through simple reactions [122]. In addition, GO can be employed as a chemical and biological sensor, since it is capable of detecting proteins supported by biomarkers (i.e., cancer detection) and thus it can be considered as a very useful medical tool [123]. For instance, GO/alginate microcapsules were synthesized through electro-spraying, following a very simple procedure. An initial GO dispersion (6 mg/mL) was diluted with phosphate buffered saline (PBS) to obtain different concentrations (0.5, 1, 2 and 3 mg/mL) and next alginate (1 wt.%) was added in each dispersion. The prepared GO/alginate dispersions were then electrosprayed with a flow rate of 6 mL/h at 15 kV [124]. Finally, MSCs were encapsulated and exposed to extreme stress conditions during the injection process as shown in Figure 7 [125].

The researchers also investigated the biocompatibility of GO and its impact on the proliferation of MSCs in the case of exposure at different alkaline environments [125,126]. In an interesting work, rGO was coated onto Ti substrates through the meniscus-dragging deposition (MDD) method. The process endorsed a decrease in the contact angle followed by surface modification of the Ti substrates. It was observed that rGO remarkably increased the proliferation of cells after a 7-days incubation [127]. As a result, rGO-Ti substrates enhanced the ALP activity, and thus indicated a higher rate of cell proliferation [128].

In another study, it was confirmed that graphene and GO could guide the osteogenesis of MSCs. Regardless of its coating density, GO displayed upgraded cell functions in terms of cell growth, spreading, and differentiation into osteoblasts, specifically within the first two days of cultivation [129]. Recently, the adipose/bone marrow-derived MSCs were treated with graphene/GO at different concentrations (0–300 g/mL), and their viability was assessed by the AlamarBlue assay. Untreated cells were used as the positive control, while the treated ones with ice-cold methanol were used as the negative control. Finally, the fluorescence was measured using an excitation and emission wavelength at 530 nm and 580 nm, respectively [64,130].

rGO sheets and rGO nanoribbons (rGONRs) were combined with human MSCs, isolated from umbilical cord blood and both cytotoxicity and genotoxicity were assessed at various concentrations and time intervals, as demonstrated in the literature [131]. rGONRs were synthesized using MWCNTs as the precursor material. It was shown that rGONRs exhibited the same cytotoxicity at a concentration of 10 mg/mL after 1 h of exposure time as in the case of 100 mg/mL after 96 h, under continuous exposure. This fact confirmed that rGONRs could penetrate the cells and trigger DNA fragmentation and chromosomal aberrations, even at low concentrations (1 mg/mL) after 1 h of exposure time [132]. It is critical to deeply investigate and understand the mechanisms which rely on the combination of graphene-based nanostructures and stem cells due to their shape and interactions in order to mitigate any negative side effects [133].

MacDonald et al. studied the incubation of human MSCs into a graphene dispersion for 1, 5, 24 and 96 h at 37 °C [134]. The cells were detached with trypsin and centrifuged for 5 min. As negative control, the cell pellet was suspended in a Dulbecco’s Modified Eagle’s Medium (DMEM) without the presence of any graphene-based additive, while methyl methane sulfonate (100 mM) was used as the positive one. In a same manner, GONRs, GO and rGO in powder form were tested. Initially, the cell viability was evaluated by the fluorescein diacetate (FDA) method. Staining was detected using a fluorescence microscope upon mixing FDA with ethidium bromide. The surviving cells percentage was calculated by dividing the surviving cell population with the total one. Other methods have also been used to evaluate genotoxicity based on the determination of the RNA efflux. This is a comet assay which enables the detection of any DNA damage and other chromosomal aberrations, as well as the fluorescent labelling of rGONRs and cells [130,135].

As also mentioned in the introduction, Liu et al. reported on the impact of aquatic MWCNTs, GO and rGO on zebrafish embryos [30]. More specifically, zebrafish embryos were exposed at MWCNTs, purified, and subsequently oxidized by HNO_3_/H_2_SO_4_, and freshly prepared GO and rGO, at different concentrations for 96 h [11,12]. The toxicity of these carbon-based nanomaterials against the zebrafish embryos was evaluated at concentrations ranging from 0 to 100 mg/L. The results showed that rGO significantly inhibited the hatching of zebrafish embryos and decreased the length of the hatched larvae to 96 hpf. The length was similarly shrunk in the case of MWCNTs. In any case, the use of these carbon-based nanomaterials did not reveal any morphological abnormality [30].

In a similar work, the toxicity of these nanomaterials was further analysed by using a Cell Counting Kit-8 (CCK-8). More specifically, after exposure of 2 h, the CCK-8 reagent was added, and the cells were additionally incubated for 2 h. Initially, MWCNTs, GO and rGO exhibited the same toxicity against hatching and larvae length of zebrafish embryos [136]. Further, the toxicity of GO was examined in larvae and adult zebrafishes upon exposure at 0, 0.25, 0.5 and 1 mg/L for 72 h, demonstrated that the hepatotoxic phenotype was considerably reduced at the liver area with a dose-dependent decline in the number of hepatocytes [137].

**Figure 8:**
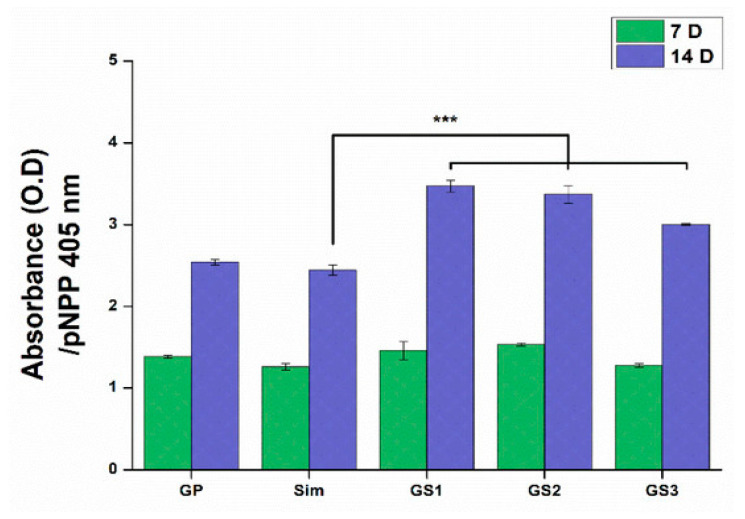
The ALP assay was performed in cells cultivated for 7 and 14 days. No significant differences were observed after 7 days, although remarkable differences were shown after 14 days when GS1 and GS2 exhibited enhanced ALP activity, while GS3 containing 1 μM of Simvastatin presented a lower level of ALP. Error bars represent +/− standard deviations (*n* ≥ 3). *** *p* < 0.001 [139].

Recently, the viability assay of embryos included the exploitation of acridine orange staining to detect cell death in live embryos. In this frame, graphene derivatives were injected to the embryo 24 h after the fertilization (hpf), while the cellular death was examined at 36 hpf. The embryos were rinsed with fish water and incubated in acridine orange (100 μg/mL) for 1 h at 28 °C. Finally, the fluorescence of the whole embryo was measured and quantified using Image Pro Plus software [138]. The use of the alkaline phosphate (ALP) assay to record the synergetic effects of Simvastatin and Polyethyleneimine inducing GO were evaluated regarding the capability for bone generation using MSCs, as presented in Figure 8 [139].

### 4.2. Graphene Oxide in Rat Mesenchymal Stem Cells

Rat MSCs have been widely explored due to their capability to regenerate and differentiate to more specialized cells, including bone cells (osteocytes), fat cells (adipocytes) and cartilage cells (chondrocytes) [140]. Bone marrow highly enables the isolation of these cells, which is mostly obtained from rat femur and tibia parts [141]. GO has been typically used in powder form, dispersed or as a coating on a substrate. Fluorescence spectroscopy is a non-destructive method to analyse molecules, structural confirmation of DNA or proteins, even at low concentrations [142]. Since GO is fluorescent, it has been widely implemented for drug delivery, antibacterial, biosensing and disease detection applications [125,143]. Very recently, Shim et al. investigated the influence of polydopamine (PDA) doped GO (PDA/GO) composites on the osteogenic differentiation via a bone morphogenetic protein receptor (BMPR) of type I and II in pluripotent ESCs and confirmed a significant enhancement [144].

Gelatine is derived from the irreversible denaturation of collagen and is used as a replacement for collagen in cell and tissue culture for biomaterial applications. Due to its similar molecular structure and functions to collagen, there has been significant progress towards the development of functional gelatine-based materials for medical devices as a result of technological advancements, such as rapid prototyping and three-dimensional printing [145]. Recently, GO–gelatine aerogels were synthesized through physical interactions. Despite having far superior structural properties to negatively charged aerogels, negatively charged aerogels outperform positively charged aerogels in terms of haemostatic activity. Hence, providing a suitable structure for the coagulation process and encourage clot formation. They are also non-cytotoxic and promote the proliferation of fibroblasts. Therefore, negatively charged GO-gelatine aerogels may be considered a potential haemostatic device for wound dressing [146]. In another study, Kluyveromyces lactis encapsulation strategies based on gelatine hydrogels were doubly cross-linked with GO and glutaraldehyde yielded in highly resistant nanocomposite encapsulate. The unique properties of GO, such as its excellent solubility and dispersibility in water and other solvents, led to its selection as a reinforcement agent. The fabricated nanocomposites had larger pore sizes, allowing for cell entrapment and proliferation, a pH-dependent swelling ratio, controllable degradation rates, and enhanced mechanical stability and integrity. As a result, these nanocomposites hold great promise for the formulation of high-performance nutraceuticals, as well as tissue engineering and high-value metabolite production [147]. Similarly, Jiao et al. developed a biodegradable rGO gelatine (rGO@Ge) composite to investigate its impact on rat adipose-derived MSCs [148]. Towards this, chondrogenic differentiation was observed by injecting kartogenin (KGN) into the stem cells proficiently. The optimum amount of KGN to stimulate proliferation and chondrogenic differentiation of the adipose-derived stem cells by a sequence of experiments was 1 μM. In addition, it included a range of markers, such as immunofluorescent (IF), toluidine blue (Tb), alcian blue (Ab) and PCR quantitative analysis of the chondrogenic markers. The results revealed that rGO@Ge could serve as a biocompatible nanocarrier to deliver KGN into the adipose-derived stem cells employing a pro-chondrogenic effect [82]. Due to this fact, great attention was paid to evaluate the performance of: (i) the proliferation capability of the cells, (ii) the recovery of the MSCs originated by rat bone marrow and (iii) the differentiation potential to adipogenic and osteogenic lineages supported by in vitro studies [149].

In another interesting study, the capability of GO treated with sodium hyaluronate (HY) (GO-HY) to accelerate bone healing in the tibia of rats was assessed. It was proved that GO-HY could be considered as a very promising material in the field, since the rate of bone repair at 100 μg/mL was remarkably enhanced [150]. Puah et al. fabricated a novel peptide-induced multilayer GO film to cultivate human Wharton’s jelly derived MSCs (WJMSCs). The outcome validated the osteogenic differentiation of WJMSCs onto the peptide-GO film which was significantly improved compared to the parent GO film. This novel peptide-GO film was highly biocompatible and could be directly applied for bone tissue regeneration [151]. Therefore, the exploitation of graphene derivatives has sparked the interest in tissue engineering and bone regeneration applications [152].

The above-mentioned results have been completely approved in the literature, since extensive characterization of the MSCs incorporating graphene-based nanomaterials has been carried out using niche analytical techniques, such as scanning electron microscopy (SEM), transmission electron microscopy (TEM), energy-dispersive X-ray (EDX), and Raman spectroscopy [130,135].

**Figure 9:**
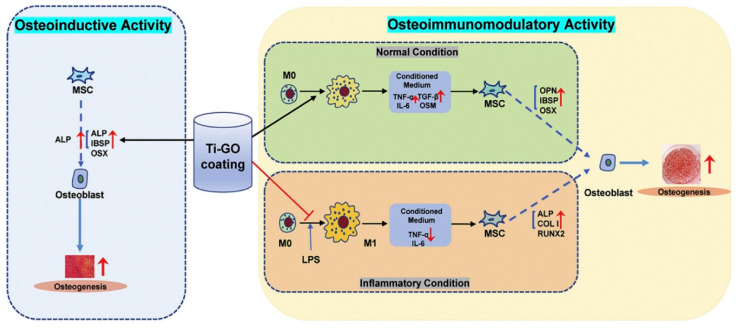
GO coatings on Ti substrate display significant biocompatibility. In osteogenic activity, GO coatings enhance osteogenic genes, osteoinductive ability and extracellular matrix mineralization of the MSCs, while GO normalizes polarization through receptors which stimulate cytokines and thus improve osteogenic differentiation [154].

ALP is an early stage osteogenic marker, whereas Alizarin red staining is a late-stage marker to identify osteogenic differentiation of bone marrow MSCs [65]. Moreover, the ALP assay has been extensively used to confirm the presence of osteoblast cells and thus the formation of new bone tissues. The MSCs markers were identified using flow cytometry, while Alizarin red was utilized for staining culture calcium deposition in tissues, whereas cell proliferation was performed according to the AlamarBlue assay. Finally, cell lysates were used to measure ALP activity after centrifugation, as illustrated in Figure 9 [153,154].

Rat bone marrow MSCs proliferation is influenced by the concentration of GO used in several treatments [155]. MSCs appeared to be highly proliferative when the concentration of GO was up to 0.1 g/mL, compared to the control group. The treatment at higher concentrations, such as 1 to 10 g/mL, resulted in a similar MSCs proliferation rate as the control group [156]. However, the cells were significantly shrunken, possibly due to the increased oxidative stress which was induced by the high concentration of GO. In support of these findings, for GO concentrations over 1 g/mL the viability of the bone marrow MSCs was slightly inhibited contrary to the cell proliferation, which was significantly reduced [157].

The management of NPs can support the impediments to enhance the benefits of cell therapy via gene delivery to the stem cells. Moreover, they may contribute to an enhanced retention of the stem cells, thus enabling the proangiogenic influence of stem cells and simulation of the extracellular matrices [97]. Previous studies have deeply investigated the chemistry of NPs and their effective role on the MSCs in terms of adhesion, proliferation, and differentiation. For instance, magnetic iron-oxide NPs were applied for labelling grafted stem cells, evaluated by magnetic resonance imaging (MRI). Likewise, chitosan-based NPs were specifically used towards the differentiation and monitoring of various types of stem cells, specifically the human MSCs [19,118]. Gelatine contains several functional groups that can be modified, and its balanced hydrophilic and hydrophobic properties aid in the loading of chemotherapeutic agents. This allows gelatine NPs to be used in anticancer drug pulmonary delivery. Co-spray drying optimized NPs with leucine were used to create nano-in-microparticles with excellent aerosolization properties. This allows for lung deposition in respirable airways, offering a promising platform for lung cancer treatment [158].

The exploitation of GO NPs intends to analyse the toxic effects as a function of the molecular mechanism of GO exposure in adult and larval zebrafishes. The results imply that the main hepatotoxic phenotype tempted by GO in zebrafish embryos was a substantial decline in the liver area and a dose-dependent reduction in the hepatocytes. Furthermore, the quantity of macrophages and neutrophils in embryos was significantly reduced contrary to the population of pro-inflammatory cytokines which was increased after the treatment with GO [137].

Similarly, gold NPs acted as striking non-viral gene vectors. In a relevant study, gold NPs were synthesized to serve as antimicrobial peptide conjugated cations and powerful nanocarriers for gene delivery to stem cells with notable antibacterial activity. The peptide took the advantage of gold NPs and commendably combined DNA and antimicrobial peptides which are crucial for the cellular reinforcement to achieve high antibacterial activity. The results showed that the peptides conjugated with gold NPs remarkably endorsed the gene transfection capability in rat MSCs. The concept of this study provided the NPs as a perfect carrier for in vivo gene activation in potential tissue regeneration systems [159]. To elaborate the role of NPs, in Table 2 the impact and effects of numerous types of NPs on rat bone marrow MSCs are listed.

GO nanosheets were tested using two in vitro biomimetic culture methods which imitated similar conditions. The former sequential-seeding method simulated the interface between GO and the established cells, whereas the co-seeding method was concerned with the interaction between GO and the migrating cells. Among them, sequential seeding is less vulnerable than the co-seeding due to the fact that cell death can be observed during co-seeding. The researchers also observed that both cell differentiation and proliferation were reliant on the concentration of pristine GO nanosheets during the in vitro culture methods [168].

A relevant study was conducted using a hydrothermal treatment of urea and sodium hydroxide for the response of rat bone marrow MSCs onto GO films. The results clearly proved that the alkali thermal treatment of GO films with urea improved biocompatibility, thus promoting the cell proliferation and increased the ALP activity. On the other hand, thermal treatment with alkali sodium hydroxide significantly enhanced toxicity, highlighting its incompatibility for cell growth [169]. It was demonstrated that osteogenic inducing medium (OIM) at concentration 0.1 g/mL increased the ALP activity and mineralized nodules, obtaining the same results, while Alizarin red staining steadily reacted with the treated groups of GO [170].

The above-mentioned studies revealed that differentiation and proliferation of the MSCs exhibit a concentration-dependent behaviour [171]. Growth factors are not solely able to promote the differentiation of MSCs into mature osteoblasts for stem cells-assisted therapy in a timely and efficient manner. In this regard, efficient techniques to promote osteogenesis are in high demand. On the other hand, GO was capable of inducing differentiation of the MSCs to osteoblasts [172]. To conclude, novel graphene derivatives are necessitated to persuade further suitability of nanomaterials among the variety of MSCs. Table 3 displays advanced GO based materials and their effect on stem cells and tissue engineering applications.

### 4.3. Current Limitations and Challenges

The use of biocompatible nanomaterials to serve as scaffolds for stem cell growth is an emerging approach towards the development and rapid progress of stem-cells mediated applications. However, there are several obstacles to be overcome before the incorporation of such materials (e.g., graphene derivatives), since their diverse role has to be further investigated.

#### 4.3.1. More Specifically

A deeper understanding of the role of graphene derivatives as a function of their physicochemical properties and underlying interaction mechanisms with the stem cells:

- The interactions between graphene and the stem cells must be defined and specified depending on the stem cells type;
- The synthetic process of graphene-based scaffolds should be highly compatible with the in vivo natural microenvironment of the stem cells;
- Despite the technological advances in nanoscale fabrication, in practise, minor progress has been made towards the development and functionalization of 3D graphene-based scaffolds, due to various scientific and technical challenges;
- In order to ensure fine control in terms of topography, size, structure, and functional groups of graphene-based scaffolds, the use of more sophisticated fabrication methods is required;
- The majority of published research on stem cell growth and proliferation control using graphene-based components has not exclusively described the disadvantages originated by the interactions within the graphene/stem cells complex;
- The long-term toxicity, performance, and biocompatibility of graphene derivatives with the stem cells should be investigated;
- Current literature lacks in vivo studies on cellular and tissue regeneration supported by graphene-based scaffolds. In this frame, certain parameters, such as biodistribution, biodegradability and biocompatibility, remain critical.

#### 4.3.2. Future Outlooks

Cytotoxicity at high doses and prolonged exposure time remains challenging for the incorporation of various graphene derivatives in biomedical applications. However, this could be overcome through simple functionalization procedures (e.g., GO treated with PEG):

- Scaffolds consisting of PEGylated GO exhibit great potential to be applied for skin, cardiac, bone and neural tissue engineering;
- The development of GO-PEG mediated technologies for clinical translation should be rapidly progressed. In addition, additive manufacturing techniques could be exploited to engineer ECM mimicked scaffolds;
- Novel PEGylated graphene derivatives to serve as active targeting agents should be further clinically investigated;
- Extensive in vivo studies should be performed in order to deeply comprehend the influence of PEGylated GO on the regenerated tissues and their biodistribution;
- The role and the long-term effect of PEGylated GO based scaffolds in tissue regeneration should be further investigated;
- Since different results could be obtained compared to the existing in vitro technology, the development of a more accurate and straightforward in vivo technology is required in order to evaluate the impact of graphene derivatives on the MSCs. This fact could also capitalize the use of graphene-based nanomaterials into more clinical applications;
- The development of a novel protein detection technology, as well as the proteins sequencing efficiency and accuracy improvement, is essential;
- A more sensitive cutting-edge technology should be established supported by advanced data collection bioinformatics in order for more details to be extracted from the current collected data.

## 5. Concluding Remarks

Graphene-based nanomaterials have been extensively used in many applications ranging from biomedicine to aerospace. This fact highlights the uniqueness of graphene, thanks to its properties, which can be incorporated in various applications to boost performance and provide improved functionality. In this context, graphene and its derivatives have recently skyrocketed interest in the emerging fields of regenerative medicine and tissue engineering, to serve as biomimetic scaffold materials for such stem cell mediated applications. Their distinct surface properties, chemical versatility, as well as the excellent tissue-specific inductive capability and biocompatibility, can be well-combined with the superior mechanical properties, to provide enhanced tissue regeneration in a controlled manner. Therefore, the potential applicability of an integrated graphene stem cell complex system in tissue engineering applications is extremely high, although new challenges will spawn in the future. Nonetheless, the effect of graphene-based nanomaterials in biomedical applications is relatively unexplored and thus its potential incorporation could contribute great achievements to the field of regenerative medicine.

## Figures and Tables

**Table 1:** Exposure of various cell types to graphene derivatives in different conditions.

Graphene-Based Nanomaterials (Exposure Conditions)	Cell Types	Effects	Ref.
GO [50 µg/mL for 24 h]	Human fibroblast cells	Dose and time dependent cytotoxicity, causes cell floating and apoptosis	[113]
GO [1–100 µg/mL for 24 h]	Human monocyte-derived macrophages	Impact on cellular generation and promotion of Reactive oxidative stress (ROS)	[114]
GO [50 µg/mL for 24 h]	Mouse embryonic fibroblasts	Higher degree of cytotoxicity and apoptosis	[115]
Carboxyl Graphene nanoplates [4 μg/mL for 24 h]	Human liver carcinoma cell (HepG2)	High cytotoxicity and induction of ROS	[116]
rGO [50 μg/mL for 24 h]	HepG2	High cytotoxicity and induction of ROS	[117]

**Table 2:** Effects of various NPs on Rat Bone Marrow MSCs.

NPs	Effects on Rat Bone Marrow MSCs	Ref.
Gold	Efficient coating for gene delivery to MSCs with antibacterial activity	[159]
Calcium phosphate ceramic	Commonly applied in bone tissue engineering to present higher cell viability and cell adhesion	[160]
Gelatine-based hydrogels	Enhancement of rat neonatal cardiomyocyte adhesion and stimulate maturation	[161]
Poly-L-lactide (PLLA) scaffold	From thermal-induced phase separation techniques to enhance the regeneration of bone marrow MSCs and to increase calcium deposition	[162]
Self-supporting graphene hydrogel (SGH)	Implanted into subcutaneous sites of rats leading to the formation of new blood vessels stimulating osteogenic differentiation	[163]
Magnetic GO	Exhibited a significant increase in bone formation related genes such as β-catenin, Runx2, BMP-2, and OCN	[129,164]
Titanium	Tends to release BMP2 differentiation inducing proteins to increase osteogenic in vitro and in vivo differentiation	[149,165]
Aluminium oxide	Hippocampal cells were subjected to severe toxicity and apoptosis. Adipose-derived MSCs suppressed oxidative stress and stimulated immunity, as well as alleviated toxicity of Al_2_O_3_ via the regulation of P53, Aβ, SOX2, OCT4, and CYP2E1 signalling in hippocampal cells	[166]
Selenium	The combination of Se NPs and stem cells greatly reduced Aβ deposition while the concentration of brain derived neurotrophic factor (BDNF) was increased. Accordingly, excellent results in neuroprotection of Alzheimer’s disease were obtained	[167]

**Table 3:** Summary of GO based nanomaterials in MSCs.

Nanomaterials	Parameters	Outcomes	Applications	Ref.
GO/alginate	Addition of 0.05 to 1.0 mg mL^−1^ GO to 3% alginate	3D scaffolds printed with MSCs and alginate/GO greatly improved osteogenic differentiation	Bone regeneration	[173]
Graphene		No evidence of cytotoxicity in stem cell cultures Promoted cardiomyogenic differentiation	Stem cell engineering	[174]
GO/alginate	2 mg/mL of GO and 20 mg/mL of alginate	Based on in vitro studies, MSCs viability increased under oxidative stress conditions with H_2_O_2_ In vivo studies also revealed enhanced therapeutic efficacy of MSCs delivery in r(GO/alginate) microgels	Tissue regeneration	[124]
GO		Drug-GO complex loaded on MSCs demonstrated selective killing of cancer cells without affecting the MSCs viability	Platform for drug delivery	[74]
Graphene nano-onions (GNOs, GONRs, and GONPs)	Concentrations ranging from 5 to 300 µg/mL	There were no significant differences in cytotoxicity between graphene nanostructures with less than 50 µg/mL concentrations and untreated controls Low (10 µg/mL) or high (50 µg/mL) graphene concentrations had no effect on adipogenic and osteogenic differentiation potential of MSCs	MSCs-based imaging and therapy	[64]
Graphene-incorporated chitosan substrate	0, 0.05, 0.5, and 5% *w*/*w* rGO	Promoted adhesion and human MSCs differentiation	Tissue engineering	[175]
GO incorporated cellulose acetate (CA)	0 to 1 wt.% of GO	Biomineralization and human MSCs osteogenic differentiation were improved significantly	Bone tissue engineering and regenerative medicine	[176]
GO-calcium phosphate	0.5 μg mL^−1^ GO and 10 μg mL^−1^ calcium phosphate	Synergistic osteoinductive effect on human MSCs	Bone tissue engineering and regenerative medicine	[177]
Graphene-based nanomaterials		Boosted the effective dose of MSCs-Exos at local wound sites. Enabled MSCs-Exos to achieve improved long-term acting time, retention rate, and stability	Tissue engineering	[178]
GO		The use of peptide and protein-GO conjugates: Stem cell growth Increases cytocompatibility Transmits chemical signals that promote MSCs differentiation through a specific pathway	Tissue engineering	[179]
Cross-linked polyethylenimine (PEI) grafted GO		Neuronal differentiation of MSCs with function was significantly accelerated both in vitro and in vivo	Regenerative therapy	[180]
Graphene/polycaprolactone scaffolds	1, 3, 5 and 10 wt.% of graphene	MSCs did not react toxically to composite robocast scaffolds Cells proliferate and differentiate well on scaffold surfaces	Cartilage tissue engineering	[181]
Silk fibroin and GO	0, 0.05, 0.1, 0.2 and 0.4 wt.% of GO	Resulted in better growth capability, proliferation, and osteogenic differentiation of cells.	Bone tissue engineering	[98]
GO		3D methacrylated gelatine (GelMA) scaffolds enhanced human MSCs osteogenesis both in vitro and in vivo	Bone regeneration	[73]
Gold nanostructure/peptide-nanopatterned GO		Successfully guiding of the human adipose-derived MSCs osteogenesis	Bone regeneration	[182]
Polycaprolactone (PCL)/GO		PCL/GO-Dex scaffold enhanced the bone differentiation and MSCs biomineralization responses	Bone tissue engineering	[183]
Graphene		Graphene was proved to be: Cytocompatible Osteogenic differentiation inducing Recognized as biomimetic in vitro substrates by human MSCs for osteogenic cell culture experiments	Bone regeneration	[184]
GO and rGO		Promoted the cardiomyogenic and angiogenic differentiation capacity of MSCs in vitro	Tissue regeneration	[185]
Silica magnetic GO (SMGO)		Improved the hepatoprotective effects of the MSCs derived condition medium on acute liver damage	Cell regeneration	[186]
Bacterial cellulose/graphene (BC/G)		Results showed that 3D-BC/G scaffold: Supported NSC growth and adhesion Maintained NSCs stemness and enhanced their proliferative capacity Induced NSCs to selectively differentiate into neurons	Neural tissue engineering	[187]
Graphene/poly(dimethylsiloxane)		Significantly promoted the stem cell proliferation	Cell therapy	[188]
3D graphene foams		Produced 3D scaffold suitable for MSCs adhesion, growth, and differentiation into DA neurons	Tissue engineering	[189]
GO		GO substrate has potential as a biomaterial for culturing Wharton’s Jelly-MSCs	Stem cell engineering	[190]
Gelatine/graphene		Gelatine conduits’ 3D microstructural and mechanical properties aided MSCs attachment and growth. Electrical stimulation within the 3D gelatine matrix improved differentiation and paracrine activity	Nerve regeneration	[191]
Graphene foam (GF)/laminarin hydrogel (LAgel)		Enhanced scaffold toughness Provided a carrier to realise the biosignals cargo to regulate cell behaviour	Tissue engineering	[192]

## Data Availability

Not applicable.
